# Effect of statin add-on therapy on cardiovascular mortality

**DOI:** 10.3389/fcvm.2024.1308695

**Published:** 2024-03-13

**Authors:** John R. Nelson, Viet Le, Jeffrey L. Anderson, Nicole Ciffone

**Affiliations:** ^1^ California Cardiovascular Institute, Fresno, CA, United States; ^2^ Intermountain Heart Institute, Intermountain Health, Salt Lake, UT, United States; ^3^ Department of Physician Assistant Studies, College of Medical and Health Professional Science, Rocky Mountain University of Health Professions, Provo, UT, United States; ^4^ Arizona Center for Advanced Lipidology, Tucson, AZ, United States

**Keywords:** cardiovascular mortality, bempedoic acid, eicosapentaenoic acid, ezetimibe, icosapent ethyl, proprotein convertase subtilisin/kexin type 9 inhibitors

## Abstract

**Introduction:**

Cardiovascular (CV) disease remains a leading cause of mortality despite statin therapy. Statin add-on lipid-lowering therapies have been investigated for CV risk reduction, but their effect on CV mortality has not been reviewed.

**Methods:**

This review describes CV outcomes trials of add-on therapies to statins, highlighting findings related to the primary composite CV endpoints and the more patient-centric endpoint of CV-related mortality.

**Results:**

Add-on ezetimibe met its primary composite CV endpoint vs. statin alone (*P *= 0.016); however, the individual endpoint of death from CV causes did not differ between groups. Add-on therapy with proprotein convertase subtilisin/kexin type 9 inhibitors achieved the primary composite CV endpoints in the respective CV outcomes trials for alirocumab (*P *< 0.001) and evolocumab (*P *< 0.001); however, neither CV outcomes trial found a difference vs. placebo in CV-related mortality. In its CV outcomes trial, icosapent ethyl added to statin therapy significantly reduced the occurrence of the primary composite CV endpoint (*P *< 0.001) and the individual endpoint of risk of CV-related death (*P *= 0.03) vs. placebo. A CV outcomes trial of bempedoic acid monotherapy achieved its primary composite CV endpoint vs. placebo (*P *= 0.004) but not the endpoint of death from CV causes.

**Discussion:**

Statin add-on therapies achieved their CV outcomes trial composite CV endpoints. Proprotein convertase subtilisin/kexin type 9 inhibitors and icosapent ethyl have approved indications for CV risk reduction. Only add-on therapy with icosapent ethyl demonstrated a significant reduction in CV mortality in the overall intent-to-treat population, possibly due to the unique pleiotropic mechanisms of eicosapentaenoic acid independent of lipid-lowering effects.

## 1 Introduction

Cardiovascular disease (CVD) is the leading cause of death worldwide, accounting for 32% of deaths (1). Globally, 17.9 million CVD-related deaths occurred in 2019, most (85%) due to myocardial infarction (MI) and stroke (1). In the United States, heart disease was the leading cause of death in 2021, with an age-adjusted death rate of 173.8 per 100,000 persons, and stroke was the fifth leading cause of death with a rate of 41.1 per 100,000 persons (2).

For more than 3 decades, the lowering of low-density lipoprotein cholesterol (LDL-C) levels has been the primary treatment target for primary and secondary prevention of cardiovascular (CV) events, with statins at the forefront of CV risk reduction (3, 4). Despite achievement of LDL-C targets with high-dose statins, residual CV risk, including CV-related mortality risk, persists for many patients (5–7). Thus, adjunctive therapy to statins to achieve additional CV risk reduction is an area of clinical and research interest. Several novel, add-on lipid-lowering therapies have been approved in the post-statin era, including ezetimibe, proprotein convertase subtilisin/kexin type 9 (PCSK9) inhibitors, icosapent ethyl (IPE), and bempedoic acid (8–12). The potential for CV risk reduction with these novel therapies has been investigated in landmark CV outcomes trials (13–17).

Cardiovascular outcomes trials typically assess efficacy using time-to-event analysis with the primary composite endpoint defined as time from randomization to time of first occurrence of any component of the composite endpoint (18). For example, a composite endpoint such as a major adverse cardiac event could include CV-related death or reinfarction, target vessel revascularization for ischemia, or stroke (18). From methodologic and regulatory standpoints, composite endpoints are pragmatic because they help ensure that sample sizes are not prohibitively high and durations of follow-up are not unreasonably long (18, 19). Use of composite endpoints increases the numbers of events that are captured and, consequently, increases statistical power and precision (19). A downside of using composite endpoints is that inclusion of components that may be less affected by the treatment can dilute or mask true treatment effects (18, 19); likewise, treatment effects on individual components of the composite endpoint may not be readily discerned. Although composite CV endpoints are useful in research, they are not particularly “patient-centric” and may not accurately reflect what patients deem to be the most important outcome(s) of an intervention (20, 21). We considered that the impact of add-on therapy on CV-related mortality [encompassing sudden cardiac death or death due to either acute MI, heart failure, stroke, CV procedure, CV hemorrhage, other CV causes (22)] may be a more patient-centric endpoint than a composite CV endpoint that encompasses CV-related mortality along with nonfatal CV events and CV procedures.

This brief review discusses CV outcomes trials of novel add-on therapies to statins, highlighting evidence for overall CV risk reduction and for CV-related mortality risk reduction.

## 2 Methods

A search of the contemporary literature (2013–2023) was conducted in PubMed/MEDLINE and clinicaltrials.gov to identify clinical trials that investigated the endpoints of CV risk reduction and CV-related mortality risk reduction among patients treated with add-on therapy to statins. Articles were limited to those in English. The keywords for the literature search were: cardiovascular outcomes, statin therapy, and cardiovascular mortality. A total of 12 clinical trials were identified between 2013 and 2023 and discussed in this review.

## 3 Cardiovascular outcomes trials of statin add-on therapy

### 3.1 Ezetimibe

Ezetimibe, a selective inhibitor of intestinal cholesterol absorption, was first approved by the US Food and Drug Administration (FDA) in 2002 as a lipid-lowering agent, with indications that included adjunctive therapy to diet for reduction of elevated total cholesterol, LDL-C, and apolipoprotein B levels in patients with primary hyperlipidemia alone or in combination with a statin (23, 24). Subsequently, lipid-lowering indications were further expanded, including for reduction of non–high-density lipoprotein cholesterol levels alone or in combination with statins in patients with primary hyperlipidemia or in combination with fenofibrate in patients with mixed hyperlipidemia (23).

The landmark Improved Reduction of Outcomes: Vytorin Efficacy International Trial (IMPROVE-IT) was a double-blind, event-driven, randomized study of 18,144 patients aged 50 years or older who had been hospitalized for acute coronary syndrome (acute MI; high-risk unstable angina) within the prior 10 days (13). Patients on long-term lipid-lowering therapy were required to have an LDL-C level between 50 mg per dL and 100 mg per dL and patients not receiving long-term lipid-lowering therapy were required to have an LDL-C level between 50 mg per dL and 125 mg per dL. Patients were randomized to receive ezetimibe 10 mg in combination with simvastatin 40 mg vs. simvastatin 40 mg and placebo to assess impact on CV outcomes over 7 years (13).

At 1 year, levels of LDL-C decreased from 93.8 mg per dL at baseline (both groups) to 53.2 mg per dL and 69.9 mg per dL in the ezetimibe plus simvastatin group and the simvastatin monotherapy groups, respectively (significant difference between groups; *P *< 0.001) (13). These LDL-C reductions were maintained over the course of the 7-year study.

The combination treatment met its primary composite endpoint, which was assessed from randomization to the first occurrence of death from CVD, major coronary event (i.e., nonfatal MI, documented unstable angina requiring hospital admission, coronary revascularization occurring ≥30 days after randomization), or nonfatal stroke (Table 1) (13). The event rate for the primary composite endpoint was 32.7% in the combination ezetimibe and simvastatin group vs. 34.7% in the simvastatin monotherapy group (HR 0.936; 95% CI, 0.89–0.99; *P *= 0.016) (13). Although ezetimibe met its primary composite endpoint of CV risk reduction, it is only approved by the US FDA for the reduction of lipid levels and is not approved for the reduction of CV risk (23).

**Table 1:** Overview of novel add-on therapies and cardiovascular risk reduction.

Agent	Study (year)	Median duration	Primary composite CV endpoint^a^^,^^b^	CV mortality endpoint^b^	Regulatory labeling for CV risk reduction
Ezetimibe (13, 23)	IMPROVE-IT (2015)	6 years	Significantly reduced in ezetimibe + simvastatin vs simvastatin group	Tertiary endpoint; not significantly different between groups	Limitations of use: Effect on CV morbidity and mortality not yet determined
Alirocumab (9, 15)	ODYSSEY OUTCOMES (2018)	2.8 years	Significantly reduced in alirocumab + statin vs placebo + statin group	Major secondary endpoint; not significantly different between groups	CV risk reduction-related indication: Reduce risk of MI, stroke, and unstable angina requiring hospital admission in adults with established CVD
Evolocumab (10, 14)	FOURIER (2017)	2.2 years	Significantly reduced in evolocumab + statin vs placebo + statin group	Other secondary endpoint; not significantly different between groups	CV risk reduction-related indication: Adults with established CVD to reduce risk of MI, stroke, and coronary revascularization
Icosapent ethyl (11, 16)	REDUCE-IT (2019)	4.9 years	Significantly reduced in IPE + statin vs placebo + statin group	Prespecified secondary endpoint; significantly reduced in IPE + statin vs placebo + statin group	CV risk reduction-related indication: Adjunct to maximally tolerated statin therapy to reduce risk of MI, stroke, coronary revascularization, and unstable angina requiring hospital admission in adults with elevated TG levels (≥150 mg per dL) and either established CVD or diabetes mellitus with ≥2 more risk factors for CVD
Bempedoic acid (12, 17)	CLEAR Outcomes (2023)	3.4 years	Significantly reduced in bempedoic acid vs placebo group	Key secondary endpoint; not significantly different between groups	CV risk reduction is not mentioned

CLEAR, Cholesterol Lowering via Bempedoic Acid, an ACL-Inhibiting Regimen; CV, cardiovascular; CVD, cardiovascular disease; FOURIER, Further Cardiovascular Outcomes Research With PCSK9 Inhibition in Subjects With Elevated Risk; IMPROVE-IT, Improved Reduction of Outcomes: Vytorin Efficacy International Trial; MI, myocardial infarction; NA, not applicable; PCSK9, proprotein convertase subtilisin/kexin type 9; REDUCE-IT, Reduction of Cardiovascular Events With Icosapent Ethyl–Intervention Trial.

^a^ Components of the primary composite CV endpoints for each CV outcomes trial: IMPROVE-IT, death from CVD, major coronary event (i.e., nonfatal MI, documented unstable angina requiring hospital admission, coronary revascularization occurring ≥30 d after randomization), or nonfatal stroke; ODYSSEY OUTCOMES, death from coronary heart disease, nonfatal MI, fatal/nonfatal ischemic stroke, or unstable angina requiring hospital admission; FOURIER, CV-related death, MI, stroke, hospital admission for unstable angina, or coronary revascularization; REDUCE-IT, CV-related death, nonfatal MI, nonfatal stroke, coronary revascularization, or unstable angina requiring hospitalization; CLEAR Outcomes, death from CV causes, nonfatal MI, nonfatal stroke, or coronary revascularization.

^b^ Intention-to-treat analysis.

**Figure 1:**
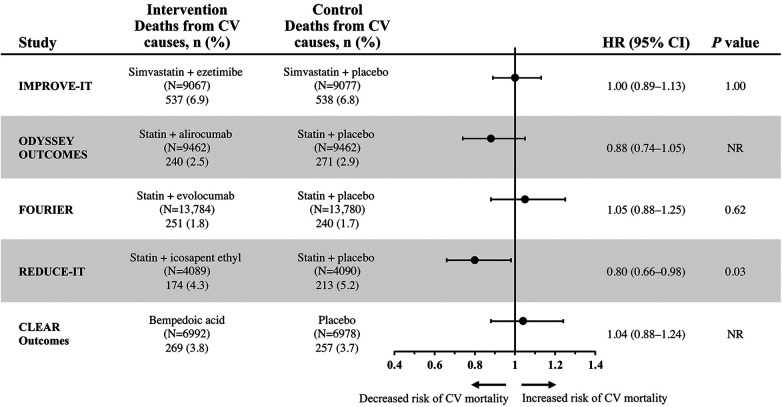
Hazard ratios of cardiovascular mortality for select lipid-lowering regimens from individual CV outcomes trials (13–17). CV, cardiovascular; NR, not reported.

Death from CV causes was a tertiary endpoint in IMPROVE-IT (13). When death from CV causes was separately considered from the primary composite endpoint, this component was not significantly different in the combination ezetimibe and simvastatin vs. statin monotherapy group (event rates of 6.9% vs. 6.8%, respectively; HR 1.0; 95% CI, 0.89–1.13; *P *= 1.00) (Figure 1) (13).

### 3.2 Proprotein convertase subtilisin/kexin type 9 inhibitors

#### 3.2.1 Alirocumab

In 2015, the FDA approved the monoclonal antibody alirocumab, a PCSK9 inhibitor, as an adjunct to diet and maximally tolerated statin therapy for treatment of adults with heterozygous familial hypercholesterolemia or clinical atherosclerotic CVD who require additional LDL-C lowering (9, 24). Subsequently, alirocumab received approval for CV risk reduction, specifically to reduce the risk of MI, stroke, and unstable angina requiring hospital admission in adults with established CVD (9). Approval of alirocumab for CV risk reduction was based on the pivotal, multicenter, event-driven, randomized, double-blind, placebo-controlled ODYSSEY OUTCOMES trial, which involved 18,924 patients aged 40 years or older hospitalized with acute coronary syndrome who were receiving maximally tolerated doses of either atorvastatin or rosuvastatin as well as add-on therapy with either alirocumab 75 mg or placebo every 2 weeks over a median of 2.8 years (15).

Mean LDL-C levels in the alirocumab add-on group were markedly reduced from 92 mg per dL at baseline to 40 mg per dL at four months, and LDL-C levels in the alirocumab group increased to 66 mg per dL by 48 months, remaining well below baseline levels; by contrast, LDL-C levels in the placebo add-on group increased to 103 mg per dL over the same time period (15). The ODYSSEY OUTCOMES trial met its primary composite endpoint, which included death from coronary heart disease, nonfatal MI, fatal or nonfatal ischemic stroke, or unstable angina requiring hospital admission (Table 1). Event rates for the primary composite endpoint were 9.5% in the alirocumab add-on group and 11.1% in the placebo add-on group (HR 0.85; 95% CI, 0.78–0.93; *P *< 0.001). Death from CV-related causes, a major secondary endpoint in ODYSSEY OUTCOMES, did not significantly differ between the treatment groups. Event rates were 2.5% in the alirocumab add-on group compared with 2.9% in the placebo add-on group (HR 0.88; 95% CI, 0.74–1.05) (Figure 1). No *P* value was reported for this CV mortality endpoint because hierarchical analysis was stopped after the first nonsignificant *P* value was observed for death from coronary heart disease (*P *= 0.38) (15).

#### 3.2.2 Evolocumab

The monoclonal antibody evolocumab, a PCSK9 inhibitor, received FDA approval in 2015 (10). Initial indications included use as an adjunct to diet added to (a) maximally tolerated statin therapy for treatment of adults with heterozygous familial hypercholesterolemia or clinical atherosclerotic CVD who require additional LDL-C lowering or (b) to other LDL-lowering therapies (e.g., statins, ezetimibe) in patients with homozygous familial hypercholesterolemia who require additional LDL-C lowering (10, 24). In 2021, an indication of CV risk reduction was added for evolocumab in adults with established CVD to reduce the risk of MI, stroke, and coronary revascularization (10). Findings from the Further Cardiovascular Outcomes Research With PCSK9 Inhibition in Subjects With Elevated Risk (FOURIER) were instrumental in helping evolocumab receive regulatory approval for CV risk reduction (10, 14).

FOURIER was a multinational, randomized, double-blind, placebo-controlled, event-driven CV outcomes trial that assessed add-on therapy with evolocumab (140 mg every 2 weeks or 420 mg monthly) vs. add-on placebo in 27,564 patients treated with statins who were aged between 40 and 85 years with clinically evident atherosclerotic CVD (i.e., diagnosis of MI or nonhemorrhagic stroke, symptomatic peripheral artery disease) as well as additional CV risk factors (e.g., age ≥65 years at randomization, current cigarette use, diabetes) for a median follow-up of approximately 2.2 years (14). Patients were required to be receiving an “optimized regimen” of lipid-lowering therapy (i.e., high-intensity statin with or without ezetimibe). In the evolocumab add-on group, median LDL-C levels decreased substantially from 92 mg per dL at baseline to 30 mg per dL within the first 12 weeks of treatment, with maintenance of this LDL-C reduction over time. Levels of LDL-C were relatively unchanged in the placebo add-on group. The primary endpoint, a composite of CV-related death, MI, stroke, hospital admission for unstable angina, or coronary revascularization, had a significantly lower event rate of 9.8% in patients in the evolocumab add-on group vs. 11.3% in patients in the placebo add-on group (HR 0.85; 95% CI, 0.79–0.92; *P *< 0.001) (Table 1) (14).

However, the individual component of CV-related death, which was one of several secondary endpoints in FOURIER, was not significantly reduced, with event rates of 1.8% in patients in the evolocumab add-on group vs. 1.7% in the placebo add-on group (HR 1.05; 95% CI, 0.88–1.25; *P *= 0.62) (Figure 1) (14).

### 3.3 Icosapent ethyl

Icosapent ethyl is a highly purified form of the omega-3 fatty acid eicosapentaenoic acid (EPA) ethyl ester that initially received FDA approval in 2012 as an adjunct to diet to reduce triglyceride (TG) levels in adults with severe hypertriglyceridemia (11, 24). Following the results of the Reduction of Cardiovascular Events With Icosapent Ethyl–Intervention Trial (REDUCE-IT) (16) in 2019, IPE received FDA approval for an indication related to CV risk reduction: as an adjunct to maximally tolerated statin therapy to reduce the risk of MI, stroke, coronary revascularization, and unstable angina requiring hospital admission in adults with elevated TG levels (≥150 mg per dL) and either established CVD or diabetes mellitus with at least 2 additional risk factors for CVD (11, 16).

REDUCE-IT was a phase 3b, multicenter, event-driven, randomized, double-blind, placebo-control led trial that compared add-on therapy with IPE 4 g vs. addition of placebo in 8179 patients treated with statins aged 45 years or older with established CVD (secondary prevention) or aged 50 or older years with diabetes and at least one additional risk factor (primary prevention) over a median of 4.9 years (16). Eligible patients had fasting TG levels 150 to 499 mg per dL and LDL-C levels 41 to 100 mg per dL while receiving a stable dose of statin for at least 4 weeks. The median LDL-C level at baseline was 75 mg per dL. Overall, 71% of the patient population had established CVD and 58% had type 2 diabetes mellitus (16).

The primary composite endpoint was a time-to-event analysis, including CV-related death, nonfatal MI, nonfatal stroke, coronary revascularization, or unstable angina (16). Add-on therapy with IPE significantly reduced the occurrence of the primary composite endpoint vs. addition of placebo, with event rates of 17.2% vs. 22% (HR 0.75; 95% CI, 0.68–0.83; *P *< 0.001) (Table 1). Notably, IPE also significantly reduced the risk of CV-related death, a prespecified secondary endpoint, with event rates of 4.3% in the IPE add-on group vs. 5.2% in the placebo group (HR 0.80; 95% CI, 0.66–0.98; *P *= 0.03) (Figure 1) (16).

### 3.4 Bempedoic acid

In 2020, the FDA approved the adenosine triphosphate citrate lyase inhibitor bempedoic acid as an adjunct to diet and maximally tolerated statin therapy for treatment of adults with heterozygous familial hypercholesterolemia or established atherosclerotic CVD who require additional lowering of LDL-C levels (12, 25). Approval was based on results from 2 pivotal phase 3, randomized, double-blind, placebo-controlled clinical trials, namely Cholesterol Lowering via Bempedoic Acid, an ACL-Inhibiting Regimen [CLEAR] Harmony (26) and CLEAR Wisdom (27). CLEAR Harmony and CLEAR Wisdom included 2,230 and 779 patients, respectively, with atherosclerotic CVD, heterozygous familial hypercholesterolemia, or both, who were receiving stable doses of maximally tolerated lipid-lowering therapy (26, 27). While these were lipid-lowering studies, both reported rates of CV-related deaths among their adverse events. No significant differences were found in CV-related mortality between the add-on bempedoic acid and placebo groups in either study (26, 27).

Recently, a CV outcomes trial was conducted to evaluate whether bempedoic acid may reduce CV risk. In the 2023, event-driven, CLEAR Outcomes trial, 13,970 patients intolerant to statins were randomized to receive bempedoic acid 180 mg or placebo daily over a median of 40.6 months (17). After 6 months of treatment, LDL-C levels were reduced from the mean baseline level of 139 mg per dL to 107 mg per dL in the bempedoic acid group, while LDL-C level was relatively unchanged at 136 mg per dL in the placebo group. The primary composite endpoint included death from CV causes, nonfatal MI, nonfatal stroke, or coronary revascularization. The CLEAR Outcomes achieved its primary composite efficacy endpoint, with significantly lower event rates of 11.7% in the bempedoic acid group vs. 13.3% of patients in the placebo group (HR 0.87; 95% CI, 0.79–0.96; *P *= 0.004) (Table 1). By contrast, death from CV causes, a key secondary endpoint, was not significantly different between groups, with event rates of 3.8% and 3.7% in the bempedoic acid and placebo groups, respectively (HR 1.04; 95% CI, 0.88–1.24) (Figure 1). No *P* value was reported for this CV mortality endpoint because hierarchical analysis was stopped after the first nonsignificant *P* value was observed for fatal or nonfatal stroke (*P *= 0.16) (17).

### 3.5 Ongoing CV outcomes trials

Clinical trials evaluating CV outcomes are currently underway for several novel add-on lipid-lowering agents. The ORION-4 and VICTORION-2 Prevent trials are ongoing to determine whether treatment with inclisiran, a small interfering ribonucleic acid that inhibits hepatic PCSK9 production, decreases the risk of CV events (28). The PREVAIL trial is currently investigating whether obicetrapib, a cholesteryl ester transfer protein inhibitor, lowers the risk of CV events in patients with CVD on maximally tolerated lipid-modifying therapy (29, 30). The impact of a reduction in lipoprotein (a) on CV outcomes is being investigated in two clinical trials: Lp(a) Horizon assesses pelacarsen, an antisense oligonucleotide, among patients with established CVD, and OCEAN(a) investigates olpasiran, a small interfering ribonucleic acid, among patients with prior MI or percutaneous coronary intervention (31–33).

## 4 Discussion

For clinicians caring for patients with established atherosclerotic CVD or with CV risk factors, knowledge that one of their patients has succumbed to a CV event brings into focus the finality of death. This stark reminder serves as a motivator to continue to strive for prevention of CV-related death. A comprehensive approach is essential and should not only optimize CV risk factors such as lipid levels, glucose levels, and blood pressure, but it should also address residual CV risk using add-on therapies.

This review found that ezetimibe, evolocumab, alirocumab, and IPE are effective, add-on, lipid-modulating therapies that can address the residual CV risk that persists in patients treated with statins. Additionally, bempedoic acid reduces CV risk in patients intolerant to statins. Each of these therapies has demonstrated significant reduction in CV risk in their respective CV outcomes trials, as measured by primary composite CV endpoints (13–17); however, when they are separately examined, the more patient-centric outcome of CV-related mortality was only significantly reduced by IPE (Figure 1) (13–17). The impact of IPE in total CVD event reduction in REDUCE-IT was substantial; for every 1,000 patients treated with IPE over 5 years, approximately 159 total primary endpoints could be prevented, including 12 CV deaths, 42 MIs, 14 strokes, 76 coronary revascularizations, and 16 hospitalizations for unstable angina (34). Unlike other add-on therapies that primarily exert LDL-C-lowering effects, it is thought that the mechanisms underlying CV risk reduction with IPE are due to pleiotropic effects of EPA that extend beyond cholesterol and TG lowering. These pleiotropic effects of EPA have been extensively reviewed and include anti-inflammatory, anti-oxidation, anti-arrhythmic, anti-thrombotic, and anti-platelet mechanisms and cell membrane stability/signaling effects (35, 36). Their importance is supported by the finding in REDUCE-IT that changes in serum EPA levels were associated with most of the observed CV risk reduction, with only minimal contribution by changes in TG, LDL-C, high-density lipoprotein cholesterol (HDL-C), non-HDL-C, apolipoprotein B, high-sensitivity C-reactive protein, and remnant lipoprotein cholesterol (35, 37, 38). Risk reductions with IPE were also similar across baseline LDL-C level categories, confirming LDL-C independent pathways are involved in its mechanism of action, in contrast to the other statin add-on agents described in this review. Of note, IPE is the only omega-3 fatty acid drug approved by the FDA to reduce CV risk, with consistent CV benefit and trends observed across studies, including REDUCE-IT (11, 16), the recent Randomized Trial for Evaluation in Secondary Prevention Efficacy of Combination Therapy–Statin and Eicosapentaenoic Acid (RESPECT-EPA) (39), and in an earlier EPA trial, the Japan EPA Lipid Intervention Study (40). Utilizing REDUCE-IT US event rates, FDA eligibility criteria, and National Health and Nutrition Examination Survey data, an estimated 4.6 million US adults would be eligible for IPE and 27,377 CV deaths would be prevented over 4.9 years (41, 42).
